# Uncovering Molecular Quencher Effects on FRET Phenomena in Microsphere-Immobilized Probe Systems

**DOI:** 10.1021/acs.analchem.3c01064

**Published:** 2023-08-31

**Authors:** Mary Catherine Adams, Valeria T. Milam

**Affiliations:** ^†^School of Materials Science and Engineering, ^‡^Parker H. Petit Institute for Bioengineering, Bioscience Georgia Institute of Technology, 771 Ferst Drive NW, Atlanta, Georgia 30332-0245 United States

## Abstract

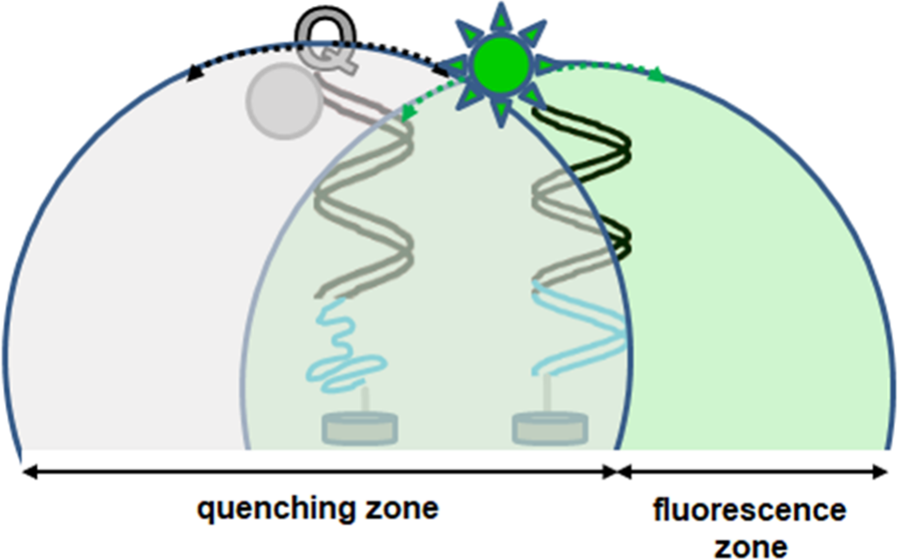

Double-stranded (ds) oligonucleotide probes composed of quencher-dye sequence pairs outperform analogous single-stranded (ss) probes due to their superior target sequence specificity without any prerequisite target labeling. Optimizing sequence combinations for dsprobe design requires promoting a fast, accurate response to a specific target sequence while minimizing spontaneous dsprobe dissociation events. Here, flow cytometry is used to rapidly interrogate the stability and selective responsiveness of 20 candidate LNA and DNA dsprobes to a 24 base-long segment of severe acute respiratory syndrome coronavirus 2 (SARS-CoV-2) RNA and ∼243 degenerate RNA sequences serving as model variants. Importantly, in contrast to quantifying binding events of dye-labeled targets via flow cytometry, the current work employs the Förster resonance energy transfer (FRET)-based detection of unlabeled RNA targets. One DNA dsprobe with a 15-base-long hybridization partner containing a central abasic site emerged as very stable yet responsive only to the SARS-CoV-2 RNA segment. Separate displacement experiments, however, indicated that ∼12% of these quencher-capped hybridization partners remain bound, even in the presence of an excess SARS-CoV-2 RNA target. To examine their quenching range, additional titration studies varied the ratios and spatial placement of nonquencher and quencher-capped hybridization partners in the dsprobes. These titration studies indicate that these residual, bound quencher-capped partners, even at low percentages, act as nodes, enabling both static quenching effects within each residual dsprobe as well as longer-range quenching effects on neighboring FAM moieties. Overall, these studies provide insight into practical implications for rapid dsprobe screening and target detection by combining flow cytometry with FRET-based detection.

Reliable and rapid oligonucleotide sensing platforms are key to assuring food and water safety^1,2^ and assessing disease and infection status of individuals and populations.^3−6^ Förster resonance energy transfer (FRET) is a versatile fluorescence-based sensing approach offering sensitive and wash-free detection of unlabeled oligonucleotide targets. One classic paradigm involves molecular fluorescent donor and acceptor (e.g., quencher) species with suitable spectral overlap. Here, each discrete donor–acceptor pair has a one-to-one correspondence with each other and a given target species.^7,8^ The distance-dependent response involving nonradiative energy transfer between an acceptor–donor pair enables FRET as a “spectroscopic ruler”^9,10^ to infer likely atomic spatial arrangements within a secondary structure. While not detailed here, FRET studies often incorporate a different fluorophore instead of a quencher as the acceptor. In such cases, spectral shifts in emission events rather than signal off–on events occur as dynamic conformational changes adjust the separation distance between donor and acceptor species. Studies of self-folded hairpins or molecular beacons^11,12^ and dsprobes typically involve fluorescence spectroscopy of oligonucleotide solutions initially in a signal-off state. Here, the initial secondary structure promotes the close proximity of quencher and dye species, resulting in static quenching. Ideally, a specific oligonucleotide target hybridizes to the molecular beacon, causing a hairpin-to-duplex transition or to the dsprobe, causing a primary duplex-to-secondary duplex transition. Both of these transitions can sufficiently separate the quencher from the fluorescent dye to allow fluorescence to occur. Challenges for molecular beacons include their susceptibility to unfolding in the absence of target as its self-hybridized stem undergoes spontaneous “fraying and peeling”^13^ or transient melting^14^ resulting in high background fluorescence. To reduce background fluorescence, multiple acceptors can be conjugated to each molecular beacon.^15,16^ To circumvent undesirable molecular beacon sequestration to the nucleus,^17^ past *in vitro* studies^18,19^ used the tetrameric protein, streptavidin, as a successful carrier to preserve each molecular beacon as a cytosolic probe. Importantly, excess streptavidin reportedly^18,19^ favored occupation of only one of its four biotin-binding sites by an individual molecular beacon.

For dsprobes, the primary-to-secondary duplex transition is due to the displacement of the quencher-capped hybridization partner by the target. Depending on the dsprobe design, one or more displacement events can be required prior to reaching the signal-on state, historically with DNA targets,^20−24^ but with recent extensions to RNA targets.^21,25^ A single-stranded toehold in the original dsprobe favors displacement by enabling the target to nucleate and then complete secondary duplex formation.^5,22,26−31^ Real-time or quantitative polymerase chain reaction (qPCR) for DNA targets and reverse transcriptase or RT-qPCR for RNA targets can increase the copy number of unlabeled targets with accompanying proportional increases in fluorescence signal. During qPCR, a longer template sequence hybridizes to a dual-labeled short partner strand up to ∼30 nucleotides in length.^32^ Though separated by a larger end-to-end distance than self-hybridized molecular beacons, FRET initially still dominates between the molecular quencher on one end, typically the 3′ end, and the fluorescent dye on the opposing end of the short hybridization partner to maintain a signal-off state. As with molecular beacons, multiple quenchers for a given fluorescent dye can be incorporated to improve FRET efficiency and reduce background fluorescence in these asymmetric duplexes.^33^ Unlike displacement events involving exchange of partner sequences, the enzymatic degradation of dual-labeled hybridization partners occurs during amplification to allow fluorescence events. Commonly referred to as Taqman probes, these sequences require careful design to, for example, accommodate sequence variability in viral samples.^34−36^ Thus, target oligonucleotide detection through qPCR requires additional reagents, such as enzymes for amplification and selective probe degradation.

The above studies all employed soluble probes to detect the presence of selected target sequences. Particle-immobilized probes, however, provide additional advantages. A metallic nanoparticle, for example, can be employed as an efficient quenching sink for multiple fluorescent dye-capped oligonucleotides. Once target hybridization occurs, the initially quenched hairpin^37^ unfolds to induce fluorescence. Alternatively, initially quenched, nanoparticle-immobilized dsprobes release the now fluorescent hybridization partner into the surrounding solution.^38,39^ Solution studies with 200 nM to 1 μM target present and *in vitro* cell studies^38,39^ have reported a ∼2–6-fold increase in fluorescence signal attributed to RNA displacement activity on gold nanoparticle-based “nanoflares.” These potential cellular probes,^40^ however, have drawbacks. For example, gold nanoparticle-based nanoflares are susceptible to false positives due to competitive exchange of thiolated DNA probes with natural biothiols^41,42^ such as glutathione.^43^ Commercial polystyrene microspheres, on the other hand, offer covalent^44,45^ and noncovalent^46,47^ coupling routes for immobilizing probe sequences, facile centrifugation steps for target capture and separation, and multiple characterization tools^48−50^ to assess hybridization activity. In contrast to nanoflares described above, here, flow cytometry of microsphere suspensions measures the fluorescence intensity of the detected scattering centers, namely, the microspheres. In past studies^45,51−53^ of toehold-mediated displacement activity in microsphere suspensions, the Milam group used high throughput flow cytometry to quantify the displacement of FAM-labeled hybridization partners by unlabeled targets. This signal on–off detection approach allowed for both quantitative end point analysis^45,51−53^ as well as *in situ* analysis^54,55^ of displacement activity. As an alternative approach to this single dye, signal on–off approach by the Milam group, Liu et al. reported FRET-based detection of unlabeled DNA targets using microspheres functionalized with initially quenched DNA dsprobes.^56^ Depending on the DNA target concentration (up to 600 nM), their flow cytometry data indicated up to a 10^2^-fold increase in fluorescence intensity. Here, we further explore this FRET-based approach to uncover how sequence choices and biotin–streptavidin immobilization affect the stability, selectivity, and sensitivity of microsphere-immobilized dsprobes to various RNA targets. While one DNA dsprobe system emerged as highly stable yet specifically responsive to severe acute respiratory syndrome coronavirus 2 (SARS-CoV-2) RNA, clear and surprising trends in the resulting fluorescence activity are evident across the sequence parameter space explored here.

## Experimental Section

All DNA, LNA, and RNA sequences were purchased from Integrated DNA Technologies (Coralville, IA), with standard desalting. For data in Figure S1a,b in Supporting Information to identify optimal probe system(s), each immobilized probe possessed a 6-carboxyfluorescein (FAM) moiety at the 5′ end, and each hybridization partner possessed an Iowa Black fluorescence quencher (Q) moiety on the 3′ end. Stock oligonucleotides solutions were prepared and stored in TE pH 8.0 (Sigma-Aldrich, St., Louis, MO) at 100 μM. Prior to coupling to microspheres, probes and their hybridization partners were annealed by heating the solutions (2:1 quencher-capped hybridization partner to FAM-labeled biotinylated probes) to 94 °C for 2 min and then slowly cooling to room temperature. For microsphere coupling, working suspensions of 1.05 μm diameter (for all data in the Supporting Information) and 3.00 μm diameter (for all data in the main manuscript) streptavidin-coated microspheres (Bangs Laboratory, Fishers, IN) were prepared by diluting stock from 1 to 0.1% w/v in wash buffer 20 mM Tris (Sigma-Aldrich), 1 M NaCl (Sigma-Aldrich), 1 mM EDTA (Promega, Madison, WI), and 0.0005% Triton X-100 (Sigma-Aldrich), per Bangs Product Data Sheet 721. Prior to oligonucleotide coupling, microspheres were washed 3 times by centrifuging at 14,000*g* for 3 min, aspirating supernatant, and resuspending in wash buffer. The preannealed solutions of the dsprobe systems were added to washed microspheres for a final solution probe concentration of 1 μM and agitated for 15 min at 22 °C, 800 rpm on a thermomixer. Following incubation, suspension samples were washed 3 times as described above. For displacement experiments, RNA stock was added to the washed and dsprobe-functionalized microspheres for a final solution concentration of 1 μM RNA and agitated for 15 min at 22 °C and 800 rpm on a thermomixer. Microspheres were not washed following the RNA addition. To prepare samples for flow cytometry, a 5 μL microsphere suspension aliquot was added to 1000 μL phosphate-buffered saline (PBS) (Gibco, Waltham, MA). Singlet populations were gated based on scattering plots, and 10,000 counts were recorded. Further experimental details for sensitivity, alternate displacement measurements, and various titration studies are available in the Supporting Information. Consistent with standard practice for reporting flow cytometry data, fluorescence was quantified in units of molecules of equivalent soluble fluorochrome (MESF), which was calculated from the raw voltage using Bangs Laboratory Quantum FITC-5 MESF standard beads. The average MESF/μm^2^ and standard error are reported for three suspensions prepared for each dsprobe system. MESF values were normalized to the degree of labeling for each fluorescent sequence, calculated by using spectrophotometry data.

## Results and Discussion

### Identifying Optimal Dsprobe System

**Figure 1:**
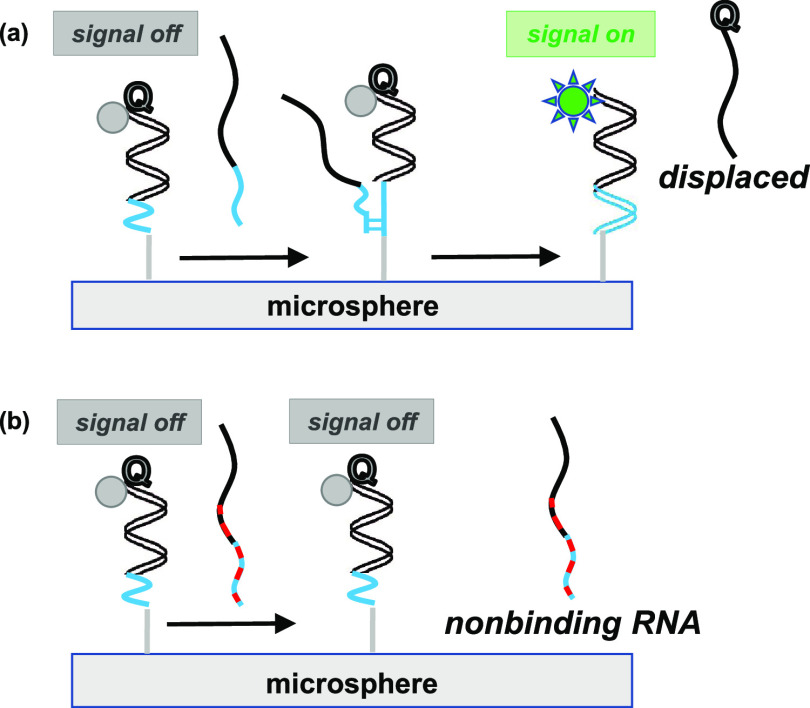
Illustrations of responsive and nonresponsive microsphere-immobilized dsprobe. (a) One dsprobe undergoes toehold-mediated displacement of the quencher-capped sequence by **SARS-CoV-2 RNA** to induce a fluorescence signal, yet (b) remains stable as a dsprobe in the presence of imperfectly matched RNA. Red indicates base mismatches in RNA. Identical colors indicate matching base segments between the probe recognition segment and RNA (black) and between the probe toehold and RNA (blue).

The current studies first screen through a series of 20 candidate locked nucleic acid (LNA) and DNA dsprobes, shown in Table S1 in the Supporting Information. This screening focuses on finding one or more dsprobes with sufficient stability prior to target addition yet with sufficient target selectivity to enable fluorescence events, as illustrated in Figure 1. To this end, chemically modified oligonucleotides such as LNA continue to gain prominence in bioscience and bioengineering fields^57,58^ as stronger hybridization partners than DNA.^59−64^ Additional rationale for the dsprobe design is provided in the Supporting Information.

First, one of two biotinylated, 24-base-long 6-carboxyfluorescein (FAM)-tagged LNA or DNA sequences (i.e., **LNA N1_FAM** or **N1_FAM**, respectively) perfectly complementary to the 24-base-long RNA target (i.e., **SARS-CoV-2 RNA**) is immobilized to microspheres. Though popular as a molecular dye, the fluorescence activity of fluorescein and its derivatives is sensitive to the nucleic acid composition and secondary structure. For example, these molecular dyes have known quenching susceptibility to a proximal guanine base^65,66^ and even to the hybridized versus unhybridized state of the dye-capped oligonucleotides.^67^ Notably, a 2022 study by Lietard et al.^68^ of all 1024 permutations of pentanucleotide sequence segments demonstrated fluorescein activity on either the 3′ or 5′ end of DNA depends on both sequence and secondary structure. Among all of the guanine-free pentanucleotides studied by Lietard et al.,^68^ 5′-FAM-ACCCX (where X=A, T, or C) sequences exhibited the lowest intrinsic fluorescence intensity. This observation is intriguingly relevant to the **N1_FAM** sequences shown in Table 1.

**Table 1:**
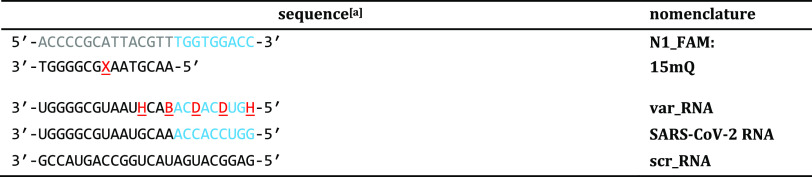
Nomenclature and Sequence of Select DNA/DNA Dsprobe and RNA Oligonucleotide Targets

a Bases are color-coded on the immobilized **N1_FAM** probe to indicate the recognition segment (gray bases) and toehold segment (blue bases). The dsprobe possesses a 5′ FAM moiety on the immobilized **N1_FAM** sequence and a 3′ quencher (Q) on its **15mQ** hybridization partner. Here, in red text, X = abasic nucleotide; B = C, G, or U; D = A, G, or U; H = A, C, or U in the mixture of model RNA sequence variants to **SARS-CoV-2 RNA**, and blue text in RNA sequences shows matching bases to the toehold segment. To facilitate comparison to the quencher-capped hybridization partner in the dsprobe, all RNA sequences are shown 3′ → 5′.

To form dsprobes in an initially signal-off state, **LNA N1_FAM** or **N1_FAM** sequences were incubated with 1 of 20 Iowa Black quencher (Q)-capped hybridization partners listed in Table S1. To find one or more suitable sequence(s), hybridization partners vary in lengths (i.e., 9 to 21 bases), chemical modifications (i.e., locked vs natural sugar backbone), and sequence fidelity (i.e., 8 out of 20 hybridization partners possess a central, nonhybridizing abasic site). These dsprobes are then conjugated to streptavidin-coated microspheres. Consistent with displacement-based strategies,^69−71^ each dsprobe has a single-stranded or initially unhybridized segment ranging from 3 to 15 bases in length next to its prehybridized recognition segment. This single-stranded segment is intended to serve as a toehold for **SARS-CoV-2 RNA** to initiate duplex formation, as illustrated in Figure 1a, while ideally preventing imperfectly matched sequences from hybridizing, as shown in Figure 1b.

To complete duplex formation with the recognition segment, the unlabeled RNA target must fully displace the quencher-capped LNA or the DNA hybridization partner. A fluorescence signal from the now unquenched **N1_FAM** should ensue from a successful toehold-mediated displacement event. A heterogeneous mixture of ∼243 similar, yet imperfectly matched, sequences (i.e., **var_RNA**) served as model variants to assess target specificity (see Table 1). Depending on the base length of the quencher-capped hybridization partner, one or more mismatches occur within each **var_RNA** segment that is intended to first hybridize to the toehold segment. Thus, as prior oligonucleotide solution experiments and theoretical studies indicate,^72,73^ the greater number of total base-pair matches in the recognition + toehold segments may still favor displacement of any shorter and/or mismatched hybridization partner. In contrast, the scrambled RNA sequence (i.e., **scr_RNA)** possesses only a few Watson–Crick base-pair matches with the probe and is thus not anticipated to participate in toehold-mediated displacement events. Flow cytometry analysis in parts a and b of Figure S1 and a more detailed discussion of all 20 candidate dsprobe systems in the absence and presence of unlabeled RNA sequences are provided in the Supporting Information.

One particular dsprobe listed in Table 1, namely, **N1_FAM:15mQ**, exhibited the best balance as a stable dsprobe that is also highly and selectively responsive to **SARS-CoV-2 RNA**. Here, **15mQ** forms an imperfect duplex with one symmetric internal loop flanked by two 7-base-long duplex segments. As shown in Figure S1b, its 9-base-long toehold allows for extensive, yet selective, displacement of **15mQ** by **SARS-CoV-2 RNA** to induce substantial fluorescence from **N1_FAM**. On the other hand, little, if any displacement, of **15mQ** by **var_RNA** occurs, as evidenced by the lack of fluorescence activity from **N1_FAM**. Thus, these model variants do not appear competitive as replacements for **15mQ** despite each **var_RNA** sequence having a greater number of total base-pair matches than **15mQ** for **N1_FAM** probe, including a continuous 11-base-long match to the recognition segment, as indicated in Table 1. Having selected the **N1_FAM:15mQ** dsprobe for further studies, we assessed the limit of detection next.

### Sensitivity Limit in Select Dsprobe System

**Figure 2:**
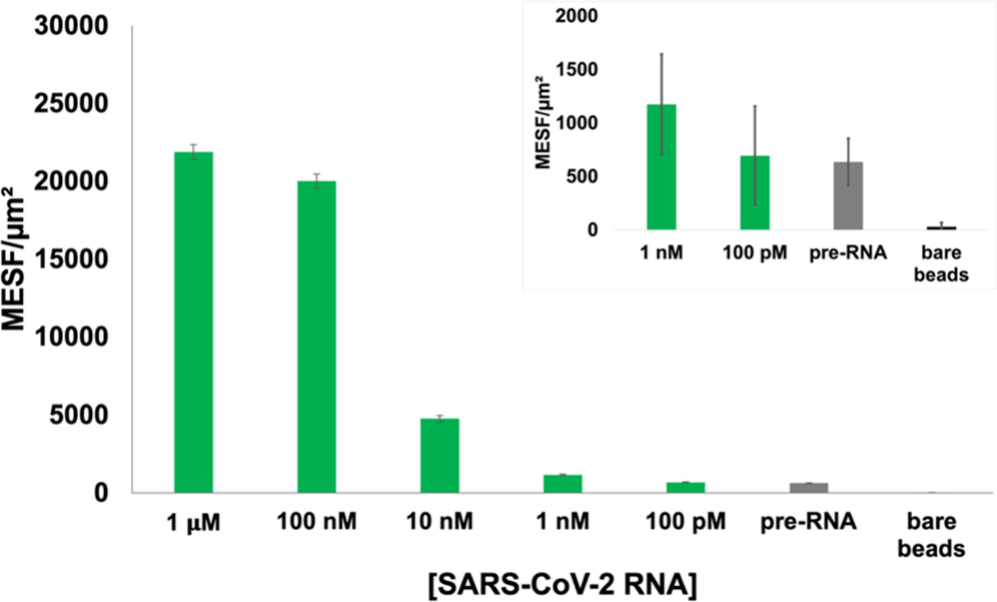
Bar graph of normalized MESF of **N1_FAM:15mQ** following the addition of **SARS-CoV-2 RNA** at concentrations ranging from 100 pM to 1 μM. The inset shows the four rightmost data sets on a smaller y-scale. Each sample condition was performed in triplicate, and error bars represent the standard deviation.

To determine the sensitivity of the optimized dsprobe to **SARS-CoV-2 RNA**, **N1_FAM:15mQ**-coupled microspheres were incubated with 100 pM to 1 μM **SARS-CoV-2 RNA**. The resulting fluorescence signals were compared to the background fluorescence of the microspheres prior to RNA addition, as shown in Figure 2. The theoretical dsprobe concentration of each suspension reaction volume (25 μL) was calculated using the manufacturer’s reported biotin-binding capacity. Further, assuming a 1:1 dsprobe to RNA target correlation, this corresponds to a concentration of 0.5 nM **SARS-CoV-2 RNA**. At higher RNA concentrations, while saturation of probes by bound RNA is possible, complete displacement of all **15mQ** is still not likely to occur unless excess target is introduced.^74^ Compared to the 1 μM RNA sample, the 100, 10, 1 nM, and 100 pM samples exhibited decreases in fluorescence signal of 9, 78, 95, and 97%, respectively, as shown in Figure 2. We wanted to next separately assess potential phenomena resulting in the marked decrease in fluorescence signal at multiple higher RNA concentrations exceeding the theoretically possible detection level of 0.5 nM. Several phenomena were hypothesized to be the cause of this discrepancy: incomplete displacement of the quencher-capped sequence; homo-FRET activity between neighboring FAM molecules; and/or longer-range quenching activity by the quencher molecules. Experiments in the next three subsections examine each of these possible explanations.

### Separate Quantification of Primary Duplex Formation and Extent of **15m** Displacement by RNA

**Figure 3:**
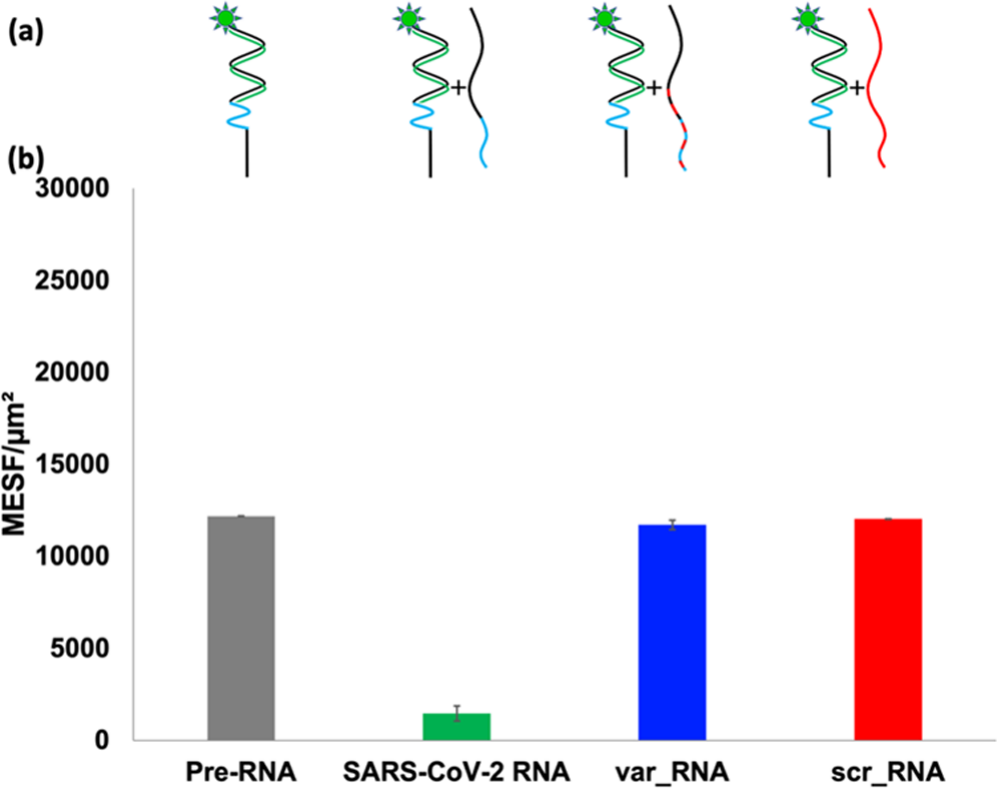
(a) Accompanying schematics of **N1:15m_FAM** duplexes immobilized on microspheres prior to (far left) and following incubation with various RNA sequences color-coded to indicate matching and nonmatching bases with immobilized **N1**; (b) bar graphs of MESF measured for **N1:15m_FAM** duplexes prior to RNA addition (gray) and following addition of **SARS-CoV-2 RNA** (green), **var_RNA** (blue), or **scr_RNA** (red) sequences. Each sample was performed in triplicate, and error bars indicate standard deviation.

In order to quantify the extent of **15m** displacement by the various RNA sequences, an experiment was conducted using unlabeled **N1** hybridized to the FAM-labeled hybridization partner, **15m_FAM**. In contrast to dsprobes initially in a quenched state depicted in Figure 1a,b, these **N1:15m_FAM** primary duplexes allow fluorescence events to occur until an event drives removal of the soluble **15m_FAM** sequence. To quantify **15m_FAM** removal in the presence of various RNA sequences, fluorescence was measured before RNA addition and then after adding **scr_RNA** and **var_RNA**, as illustrated in Figure 3a. As shown in Figure 3b, a less than 4% decrease in fluorescence occurs with either of these RNA controls. In contrast, the fluorescence decreased by 88% in the presence of **SARS-CoV-2 RNA**. Based on the overall stability of **N1:15m_FAM** in the presence of either scrambled or imperfectly matched RNA, this marked fluorescence decrease in the presence of **SARS-CoV-2 RNA** must be due to displacement of the shorter, FAM-labeled sequence. These results confirm the hypothesis that while displacement of **15m** by **SARS-CoV-2 RNA** is extensive, it is not complete. Thus, in the dsprobes composed of FAM-capped and quencher-capped duplexes, the potential for longer-range quencher effects of residual Iowa Black quencher-capped hybridization partners as well as self-quenching effects between neighboring FAM moieties was explored next.

### Titration Experiments to Determine If Self-Quenching of Immobilized FAM Occurs

Homo-FRET or self-quenching is attributed to quenching of fluorescence events due to the close proximity between identical dye molecules. For FAM, a Förster radius of 5–6 nm is reported^75^ for such self-quenching to occur, typically in a solution in which dye molecules are concentrated but freely diffusing. Based on the reported^76^ 2 nm separation distance between nearest-neighbor biotin-binding pockets on streptavidin^76^ and theoretical RNA and DNA segment lengths^77,78^ illustrated in Scheme S1a,b in the Supporting Information, an estimated separation distance of ∼2–6 nm occurs between various combinations of next neighbor sequence possibilities. Examples of nearest neighbors range from two identical upright neighboring **N1_FAM:SARS-CoV-2 RNA** duplexes to one single-stranded **N1_FAM** next to a ds**N1_FAM:15mQ** to mimic incomplete dsprobe formation prior to RNA incubation.

**Figure 4:**
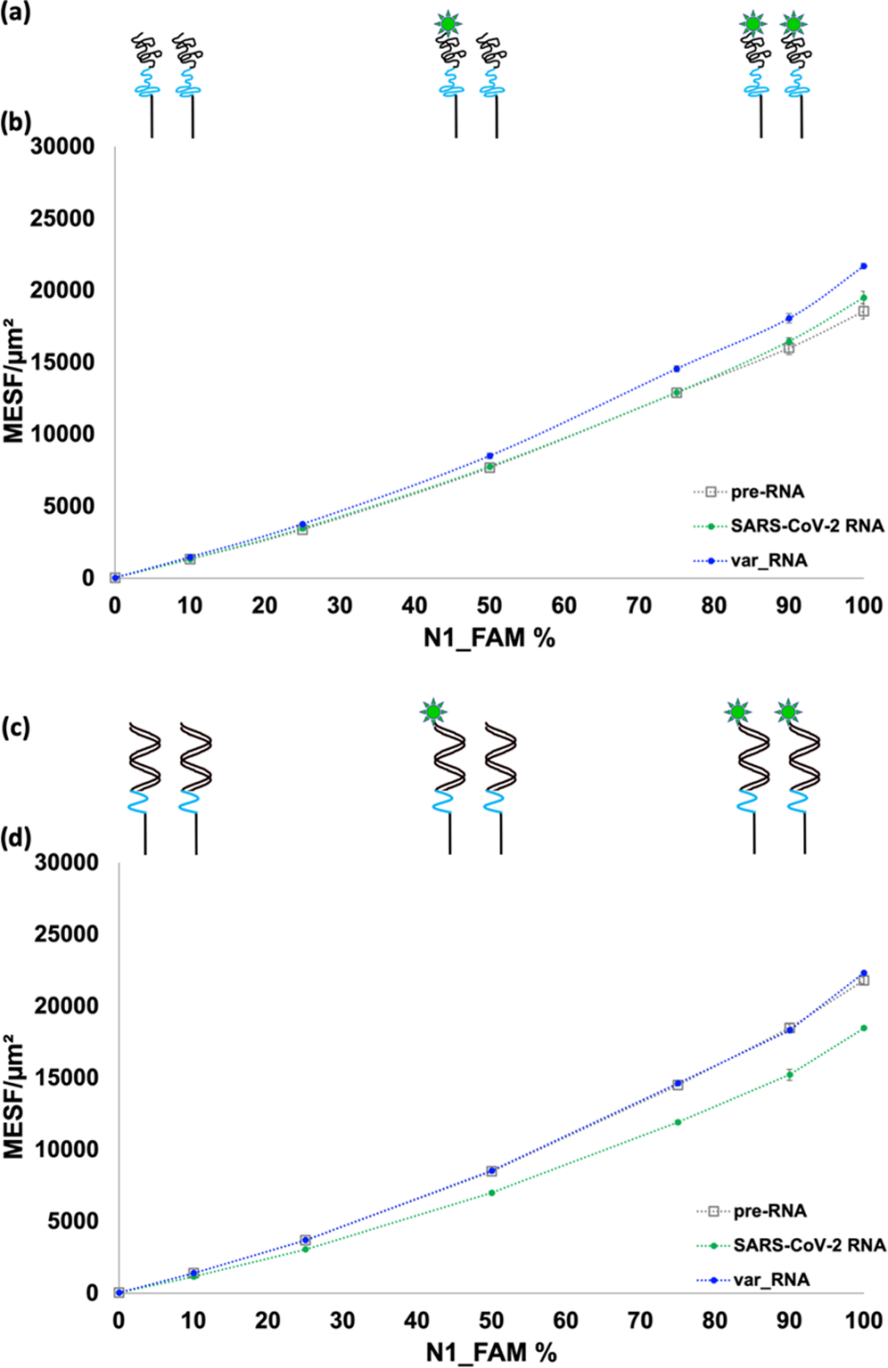
(a) Schematics of ss**N1** titrated with 0, 50, and 100% **N1_FAM** prior to RNA addition. (b) Plot of MESF of microspheres functionalized with unlabeled, ss**N1** titrated with varying percentages of ss**N1_FAM** in the absence of RNA (gray series) and following the addition of **SARS-CoV-2 RNA** (green series) and **var_RNA** (blue series). (c) Schematics of ds**N1:15m** titrated with 0, 50, and 100% ds**N1_FAM:15m** prior to RNA addition. (d) Plot of MESF of microspheres functionalized with unlabeled, ds**N1:15m** titrated with varying percentages of ds**N1_FAM:15m** in the absence of RNA (gray series) and following the addition of **SARS-CoV-2 RNA** (green series) and **var_RNA** (blue series). Samples were done in triplicate, and error bars represent standard deviation, with dotted lines added to guide the eye for each data set.

Two related studies were thus conducted in order to determine if the fluorescence response is directly proportional to the amount of **N1_FAM** in the system and if this fluorescence response is affected by the hybridization state of microsphere-immobilized **N1_FAM** sequences. As illustrated in Figure 4a, varying percentage ratios of unhybridized or ss**N1** and ss**N1_FAM** were coupled to the microspheres. Suspensions were then incubated with **var_RNA** or **SARS-CoV-2 RNA** to compare the resulting fluorescence measurements after RNA addition, as shown in Figure 4b. The pre-RNA fluorescence of the microspheres continuously increased with increasing concentrations of **N1_FAM** in the system without appearing to approach saturation, indicating that homo-FRET between nearby FAM molecules is unlikely. The addition of **SARS-CoV-2 RNA** did not result in any significant change in fluorescence overall; however, the addition of **var_RNA** resulted in a proportionally higher fluorescence signal, most evidently starting at 50% and higher **N1_FAM**.

For the second titration study, varying ratios of **N1** and **N1_FAM** prehybridized with unlabeled, unquenched **15m** were coupled to the microspheres, as illustrated in Figure 4c. Suspensions were then incubated with **var_RNA** or **SARS-CoV-2 RNA** to compare the resulting fluorescence activity before and after RNA addition, as shown in Figure 4d. Similar to the pre-RNA series for ss**N1** in Figure 4b, the fluorescence of the pre-RNA series for ds**N1** also continuously increased with increasing concentrations of **N1_FAM** in the system. The overlap of the **var_RNA** series with the pre-RNA series is attributed to the lack of displacement of **15m** by **var_RNA**. Interestingly, the addition of **SARS-CoV-2 RNA** resulted in lower fluorescence activity compared to the pre-RNA series, most evidently at 50% or higher **N1_FAM**. These results, taken with the results shown in Figure 4b, indicate that high concentrations of FAM do not appear to induce homo-FRET quenching. Unexpectedly, the measured fluorescence intensity appears to be impacted by the hybridization partner of the **N1_FAM** probe. For example, MESF values and trend lines do not overlap for imperfect **N1_FAM:var_RNA** duplexes and perfectly matched **N1_FAM:SARS-CoV-2 RNA** duplexes in Figure 4b. Similarly, MESF values and trend lines do not overlap for the initial asymmetric **N1_FAM:15m** duplexes and blunt-ended secondary **N1_FAM:SARS-CoV-2 RNA** duplex products in Figure 4d. To further confirm the effects of various hybridization partners on fluorescence measurements for the **N1_FAM** probe, a separate study was undertaken with one truncated **SARS-CoV-2 RNA** and two specific mismatched RNA sequence variants listed in Table S1. Results and discussion of the varying fluorescence are shown in Figure S2 in the Supporting Information. Notably, Lietard et al. investigated exhaustive sequence segment combinations next to the FAM moiety. In the current study, however, the bases in **N1_FAM** in each suspension are the same, and only the hybridization partner varies. While intriguing to pursue in future studies, individually testing each of the 243 sequence variants was beyond the scope of the current dsprobe study.

### Titration Experiments to Determine If Residual Quencher Species Has Longer-Range Effects on Neighboring FAM Moieties

**Figure 5:**
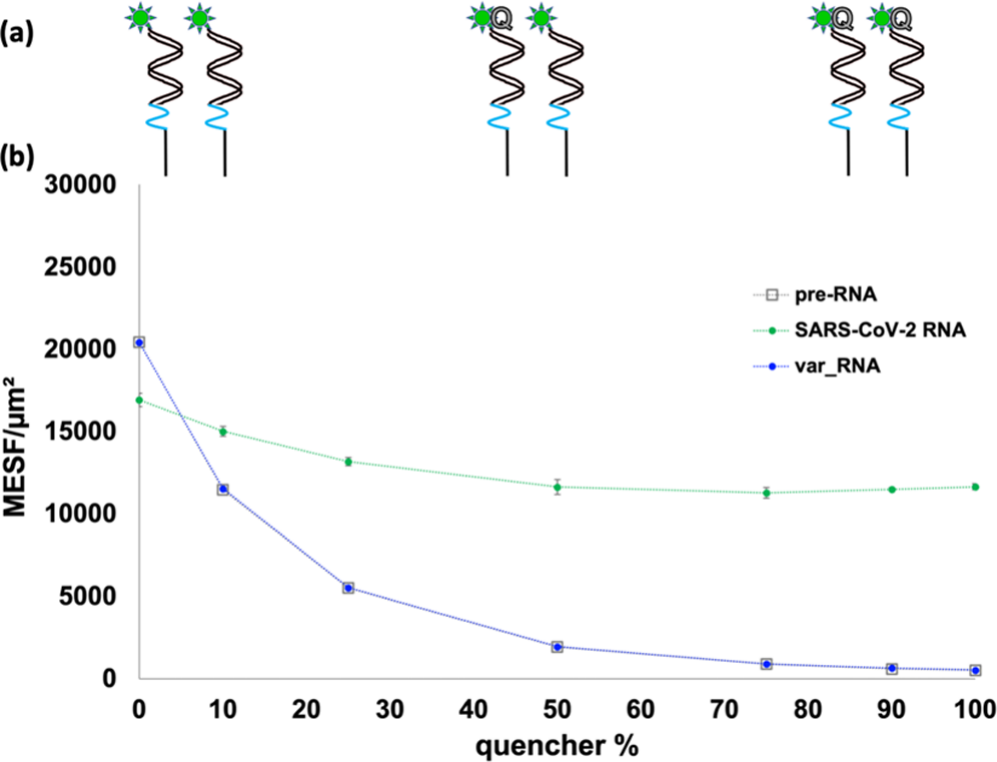
(a) Schematics of ds**N1_FAM** hybridized to a mixture of **15m_noQ** (no quencher) and **15mQ** at 0, 50, 100% ratios prior to RNA addition. (b) Plot of MESF of microspheres functionalized with ds**N1_FAM** initially hybridized to **15m** or **15mQ** at varying percentage ratios prior to RNA addition (gray series) and following the addition of **SARS-CoV-2 RNA** (green series) and **var_RNA** (blue series). Samples were done in triplicate, and error bars represent standard deviation, with dotted lines added to guide the eye for each data set.

While the previous subsection focused on fluorescence effects of FAM alone, here, the effects of neighboring Iowa Black quencher species are explored. The estimated ∼2–3 nm separation between various pairs of upright nearest-neighbor **N1_FAM** sequences on the same streptavidin (see Scheme S2b) falls below the reported^79^ Förster radius of 5.4 nm for FAM-Iowa Black quencher pairs. Thus, to determine potential longer-range effects of the Iowa Black quencher molecules, **N1_FAM** was preannealed with varying ratios of quencher-capped **15mQ** and quencher-free **15m_noQ**. These mixtures were then coupled to the microspheres and introduced to unlabeled **SARS-CoV-2 RNA** or **var_RNA**. As the pre-RNA series shows in Figure 5, the fluorescence signal disproportionately decreases with increasing concentrations of Iowa Black quencher in the system. Merely 10% **15mQ** resulted in a 44% decrease in fluorescence compared with the system with 0% **15mQ**. This particular percentage of quencher-capped hybridization partners is notable when compared to the results in Figure 3, in which displacement of **15m_FAM** by **SARS-CoV-2 RNA** was 88% complete, leaving approximately 12% of the **N1:15m_FAM** duplexes intact. Together, these results indicate that residual **15mQ** hybridization partners, even at low percentages, can disproportionately lower the fluorescence signal by quenching additional nearby FAM molecules in **N1_FAM:SARS-CoV-2 RNA** duplexes.

The addition of RNA to these dsprobe mixtures has different effects on the resulting fluorescence, as shown in Figure 5. The **var_RNA** series does not deviate from the pre-RNA series, consistent with results in Figure 3 (for **N1:15m_FAM** + **var_RNA**), indicating that **15mQ** is not readily displaced by **var_RNA**. The **SARS-CoV-2 RNA** series, however, exhibits a lower fluorescence than the pre-RNA series at 0% **15mQ**. This comparative data point for 0% **15mQ** in Figure 5 is consistent with the trends shown in Figure 4d in comparing the ds**N1_FAM** titration series before and after **SARS-CoV-2_RNA** addition. In Figure 5, starting at 10% **15mQ** and higher, however, the **SARS-CoV-2 RNA** series exhibits fluorescence higher than those of both the strongly overlapping pre-RNA and **var_RNA** series. Moreover, between 0 and 50% quencher in Figure 5, there is a 31% decrease in fluorescence (followed by a plateau at higher quencher percentages) after **SARS-CoV-2 RNA** is added. This initial decrease, followed by a plateau, is attributed to residual, undisplaced quencher-capped **15mQ** sequences. Collectively, the results of these titration studies indicate that the quenching behavior of the Iowa Black molecules extends beyond the static or contact quenching of its own hybridized FAM-capped probe. To further test this hypothesis, static quenching effects are eliminated in the final titration study by using a dual dsprobe system.

**Figure 6:**
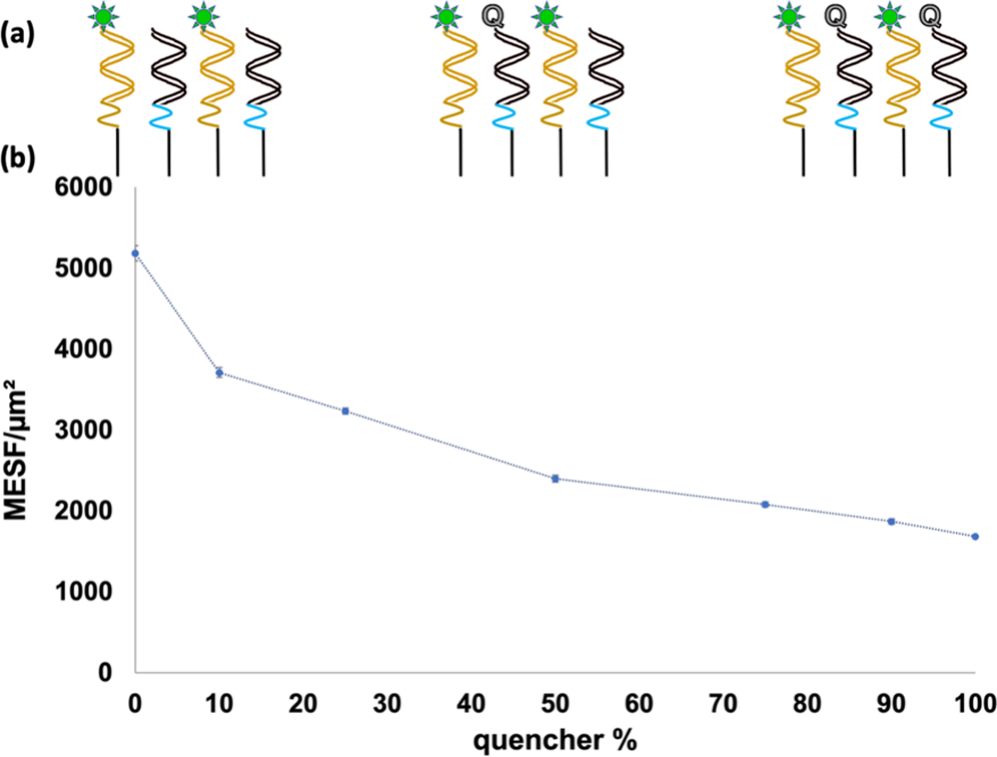
(a) Schematics of ds**A24_FAM:B15** and ds**N1** hybridized to a mixture of **15_noQ** and **15Q** at 0, 50, and 100% ratios. (b) Plot of MESF of microspheres functionalized with a 1:1 ratio of **A24_FAM:B15** duplexes to double-stranded, unlabeled **N1** probes hybridized to varying percentages of **15_noQ** and **15Q** in order to titrate the percentage of quencher species in the system. Samples were done in triplicate, and error bars represent standard deviation, with dotted lines added to guide the eye for each data set.

A final titration study was conducted using a dual duplex system in a 1:1 ratio. The first and otherwise unrelated duplex is **A24_FAM:B15**, a 24-base-long biotinylated, FAM-labeled DNA sequence and its 15-base-long DNA hybridization partner. The other duplex is unlabeled **N1** with varying ratios of quencher-capped **15Q** to quencher-free **15_noQ**. All hybridization partners are perfectly matched to their respective probe and do not exhibit any cross-reactivity (results not shown). A 1:1 mixture of preannealed **A24_FAM:B15** and ds**N1** (with varying ratios of hybridized **15_noQ** and **15Q**) was coupled to the microspheres and then interrogated via flow cytometry without any RNA additions. As shown in Figure 6, 10% **15Q** induced a 28% decrease in fluorescence signal compared to that of 0% **15Q**. As the percentage of quencher species increases, a continuous drop in fluorescence activity occurs until at 100% quencher an overall 68% decrease in fluorescence occurs. This quenching by **15Q** on **A24_FAM** confirms that quenching activity of Iowa Black can extend to a neighboring duplex, most likely its nearest-neighbor duplex on the same face of streptavidin. Consequently, for the original dsprobe system, one can infer that any residual quencher species in **N1_FAM:15mQ** can effectively dim the fluorescence of neighboring **N1_FAM:SARS-CoV-2 RNA**. Despite this longer-range quenching effect, up to a 10^2^-fold increase in fluorescence activity was observed in these microsphere-based detection platforms upon adding **SARS-CoV-2 RNA**, as shown in Figure 2.

## Conclusions

In this study, we efficiently screened numerous sequence combinations of modified and natural oligonucleotides to identify suitable dsprobes for unlabeled RNA detection using minimal chemical reagents, material supplies, and handling steps.^80^ Each suspension sample required ∼30 min of handling time for dsprobe immobilization on microspheres, followed by RNA incubation. Subsequent flow cytometry analysis of FRET events took less than 1 min. One particularly responsive dsprobe exhibited both stability and selectivity to RNA of interest with concentrations as low as 10 nM, inducing an ∼10-fold increase in fluorescence. Our studies revealed evidence of both contact quenching as well as longer-range quenching effects of residual or undisplaced molecular quencher species on neighboring FAM-capped probes. Future studies may attempt to mitigate these longer-range quenching effects by using an alternative coupling method to allow for further separation between probes. Insight from these studies combining flow cytometry with FRET analysis hold practical implications for rapid screening studies of probe candidates and for facile implementation of microsphere-immobilized dsprobes for target detection and separation as part of population surveillance such as wastewater testing^81−83^ or test kits developed in an academic laboratory setting^84^ where local resources may be limited, but timely results are essential.

## Supporting Information Available

Sequence list and data analysis of stability and response for 20 dsprobe candidates; schematics of various probe/hybridization partners with accompanying description of oligonucleotide length calculations, and fluorescence measurements of select probe/target pairs (PDF)
