# Supplementary material for: Uncovering Molecular Quencher Effects on FRET Phenomena in Microsphere-Immobilized Probe Systems
Source: Anal Chem. 2023 Aug 31;95(37):13796–803. doi: 10.1021/acs.analchem.3c01064 (PMC10515108; doi:10.1021/acs.analchem.3c01064)
Supplement: Supplementary file 1 — ac3c01064_si_001.pdf [file ac3c01064_si_001.pdf]

SUPPORTING INFORMATION

Uncovering molecular quencher effects on FRET phenomena in microsphere-immobilized probe systems

Mary Catherine Adams[1] *and Valeria T. Milam*[1,2,*]

[1]School of Materials Science and Engineering
[2]Parker H. Petit Institute for Bioengineering and Bioscience

Georgia Institute of Technology, 771 Ferst Drive NW, Atlanta, GA 30332-0245 United States

[*]Corresponding author

Table of Contents

S3 Screening candidates to select optimal dsprobe(s)
S3 **Table S1**. Nomenclature, sequence, and function of LNA, DNA, and RNA oligonucleotides employed in this work.
S4 Rationale for probe design
S6 **Figure S1**. Bar graphs of normalized molecules of equivalent soluble fluorochrome (MESF) of **(a) LNA_N1_FAM** and **(b) N1_FAM** probe systems
S7 Experimental Details
S9 End-to-end length calculations to estimate separation distance range of various probe combinations
S9 **Scheme S1. End-to-end length calculations**
S10 Effect of hybridization partner on **N1_FAM** fluorescence
S10 **Figure S2.** Bar graph of **N1_FAM** hybridized to various unlabeled hybridization partners
S11 References

Screening candidates to select optimal dsprobe(s)

The complete list of LNA and DNA microsphere-immobilized probes, their hybridization partners, and RNA targets are provided in Table S1. The immobilized sequence choice is identical to a particular DNA probe for COVID-19 from the Centers for Disease Control website as detailed in Table S1 footnotes. To find a stable, yet selectively responsive dsprobe system to toehold-mediated displacment by **SARS-CoV-2** RNA segment in Table S1, 20 different candidate quencher-capped hybridization partners were tested. The choice to incoporate a locked nucleotide at every third base position is based on displacement studies with LNA/DNA hybrids[1].

Table S1. Nomenclature, sequence, and function of LNA, DNA, and RNA oligonucleotides employed in this work.

**Sequence**[a]	**Nomenclature**	**Function**
5'-FAM-ACC^{L}CCG^{L}CAT^{L}TAC^{L}GTT^{L}TGG^{L}TGG^{L}ACC^{L}-biotin-3'	**LNA N1_FAM**	**Microsphere-immobilized probe**
3'-Q-TGG^{L}GGC^{L}GTA^{L}ATG^{L}CAA^{L}ACC^{L}ACC^{L}-5'	**LNA 21Q**	Quencher-capped LNA hybridization partners
3'-Q-TGG^{L}GGC^{L}GTA^{L}ATG^{L}CAA^{L}-5'	**LNA 15Q**	
3'-Q-TGG^{L}GGC^{L}GTA^{L}-5'	**LNA 9Q**	
3'-Q-TGG^{L}GGC^{L}GTA^{L}AXG^{L}CAA^{L}ACC^{L}ACC^{L}-5'	**LNA 21mQ**	Quencher-capped LNA hybridization partners with central abasic site
3'-Q-TGG^{L}GGC^{L}GXA^{L}ATG^{L}CAA^{L}-5'	**LNA 15mQ**	
3'-Q-TGG^{L}GXC^{L}GTA^{L}-5'	**LNA 9mQ**	
5'-FAM-ACC CCG CAT TAC GTT TGG TGG ACC-biotin-3'	**N1_FAM**	**Microsphere-immobilized probe**
3'-Q-TGG GGC GTA ATG CAA ACC ACC-5'	**21Q**	Quencher-capped DNA hybridization partners
3'-Q-TGG GGC GTA ATG CAA-5'	**15Q**	
3'-Q-TGG GGC GTA-5'	**9Q**	
3'-Q-TGG GGC GTA AXG CAA ACC ACC-5'	**21mQ**	Quencher-capped DNA hybridization partners with central abasic site
3'-Q-TGG GGC GXA ATG CAA-5'	**15mQ**	
3'-Q-TGG GGC XTA ATG C-5'	**13mQ**	
3'-Q-TGG GGX GTA AT-5'	**11mQ**	
3'-Q-TGG GXC GTA-5'	**9mQ**	
3'-UGG GGC GUA AUG CAA ACC ACC UGG-5'	**SARS-CoV-2 RNA**	RNA targets
3'-UGG GGC GUA AUG CAA -5'	**SARS-CoV-2 (15)**	
3'-GCC AUG ACC GGU CAU AGU ACG GAG-5'	**scr_RNA**	Negative RNA targets
3'-UGG GGC GUA AUH CAB ACD ACD UGH-5'	**var_RNA**	
3'-UGG GGC GUA AUA CAG ACU ACU UGA-5'	**var_3**	
3'-UGG GGC GUA AUA CAC ACA ACA UGA-5'	**var_0**	

[a]The superscript "L" indicates a locked nucleotide in select FAM-functionalized probe and quencher-capped hybridization sequences; **X** = abasic nucleotide in select quencher-capped hybridization partners; **B** = C, G, or U; **D** = A, G, or U; and **H** = A, C, or U in a mixture of model RNA sequence variants, **var_RNA**, to **SARS-CoV-2 RNA**. Each dsprobe is comprised of a 5′ FAM moiety on the immobilized sequence and a 3′ quencher (Q) on its hybridization partner. The choice of DNA probe sequence is identical to the probe sequence named **2019-nCoV_N1-P** on the Centers for Disease Control website (https://www.cdc.gov/coronavirus/2019-ncov/lab/rt-pcr-panel-primer-probes.html) accessed on February 11, 2022.

Rationale for dsprobe design and selection

The first dsprobe candidates consisted of **LNA N1_FAM** and quencher-capped LNA hybridization partners of three different lengths: 21 bases (i.e., **LNA 21Q**), 15 bases (i.e., **LNA 15Q**), and 9 bases (i.e., **LNA 9Q**). To first assess the maximum fluorescence signal from unquenched probes, Figure S1(a) shows single-stranded **LNA N1_FAM** probes alone and incubated with various unlabeled RNA sequences. As also shown in Figure S1(a) and in contrast to the relatively high fluorescence signal of unquenched probes, these three dsprobe systems all exhibited little to no fluorescence signal prior to **SARS-CoV-2 RNA** addition as well in the presence of any noncomplementary RNA. Following the addition of **SARS-CoV-2 RNA**, the longest 21 base-long LNA dsprobe showed no increase in fluorescence while the 15 base and 9 base-long LNA dsprobes showed only modest increases in fluorescence indicating little displacement by **SARS-CoV-2 RNA** occurs in these perfectly-matched LNA dsprobes. To promote additional selective displacement activity while maintaining stability, these studies were repeated using analogous quencher-capped LNA hybridization partners with a central base missing (i.e., abasic site). Probes initially hybridized to **LNA 21mQ** exhibited the same lack of fluorescence activity in the both the absence and presence of any RNA sequences. In contrast, a relatively high background fluorescence signal was found for **LNA 9mQ** indicating fewer dsprobes formed initially. While fluorescence further increased upon the addition of **SARS-CoV-2 RNA**, the increase in fluorescence also occurred in the presence of noncomplementary RNA. The most promising LNA dsprobe system employed was **LNA 15mQ** as it allowed for fluorescence signaling only in the presence of **SARS-CoV-2 RNA**; however, its observed fluorescence was significantly lower than that of single-stranded **LNA N1_FAM** in the absence of a quencher. Thus, exchange of quencher-capped **LNA 15mQ** with **SARS-CoV-2 RNA** appears incomplete. Overall, with the exception of **LNA N1_FAM:LNA 9mQ** exhibiting both higher background signal and less RNA target specificity, the affinity of LNA:LNA duplexes in the dsprobes explored here appears too high to be susceptible to extensive displacement by **SARS-CoV-2 RNA**.

Since DNA is a reportedly weaker hybridization partner compared to LNA[2-6], the stability and responsiveness of **LNA N1_FAM** hybridized to quencher-capped 9, 11, 13, and 15 base-long DNA with a central abasic site were also examined. For all but the longest of these DNA hybridization partners (i.e., **15mQ**), the background fluorescence signal and fluorescence response to negative RNA targets otherwise increased as the DNA hybridization partner base length decreased as shown in Figure S1(a) for **13mQ**, **11mQ**, and **9mQ**, respectively. While the fluorescence signal for **LNA N1_FAM:15mQ** dsprobes is nearly double that of **LNA N1_FAM:LNA 15mQ** dsprobes in the presence of **SARS-CoV-2 RNA**, it is still well below the fluorescence signal of all the remaining dsprobes possessing shorter, mismatched DNA hybridization partners (i.e., **13mQ**, **11mQ**, and **9mQ**) in the presence of **SARS-CoV-2 RNA**. Thus, while selective displacement activity appears enhanced by replacing the quencher-capped, 15 base-long, central abasic LNA hybridization partner with its equivalent DNA sequence, further improvements in detecting **SARS-CoV-2 RNA** appeared possible with stable, yet weaker affinity DNA:DNA sequence pairings in dsprobes.

Next, pure DNA dsprobes comprised of **N1_FAM** and various quencher-capped DNA hybridization partners were examined. As before with LNA dsprobes, first the fluorescence signal of unquenched **N1_FAM** before and after incubation with various RNA sequences is shown in Figure S1(b). Next, the background fluorescence signal is measured and determined to be negligible for all perfectly-matched DNA dsprobes in Figure S1(b). In comparison to the weaker fluorescence activity of perfectly-matched LNA dsprobe analogues in Figure S1(a) in the presence of **SARS-CoV-2 RNA**, a stronger dependence of fluorescence signaling on base length of **21Q**, **15Q**, and **9Q** in the presence of **SARS-CoV-2 RNA** is evident in Figure S1(b). To further explore the ability to further improve **SARS-CoV-2 RNA** detection without compromising dsprobe stability, a series of quencher-capped DNA hybridization partners with a central abasic site was then explored. Unlike the perfectly-matched dsprobes, however, a trend of increasing background fluorescence in the absence of RNA is observable for **13mQ**, **11mQ**, and **9mQ**, respectively indicating this series of imperfectly-matched dsprobes is increasingly susceptible to

spontaneous dissociation. Thus, while additional fluorescence activity in the presence of **SARS-CoV-2 RNA** does occur in each of these three imperfectly-matched dsprobe system in Figure S1(b), their lower stability broadens their susceptibility to either dissociation (likely followed by RNA hybridization to unoccupied **N1_FAM**) or to displacement by **var_RNA** sequences that resemble, but do not entirely match **SARS-CoV-2 RNA** sequences. **LNA 15mQ** and **LNA 9mQ** as hybridization partners to this DNA probe in Figure S1(b) showed similar fluorescence behavior as their sequence counterparts in Figure S1(a) (i.e., **LNA N1_FAM:15mQ** and **LNA N1_FAM:9mQ**, respectively) Thus, along with all LNA:LNA, LNA:DNA, and perfectly-matched DNA:DNA dsprobe systems discussed earlier, DNA probes initially hybridized to **13mQ**, **11mQ**, **9mQ**, **LNA 15mQ**, or **LNA 9mQ** were not further considered as optimal candidates. The chosen candidate, **N1_FAM:15mQ**, exhibited low background fluorescence and the highest fluorescence signal (27,720 MESF/μm^2) selectively in the presence of **SARS-CoV-2 RNA**. For comparison, the next best performing candidate dsprobes with low background fluorescence (in the absence of RNA) and yet selective fluorescence signal to SARS-CoV-2 were **N1_FAM:LNA_15mQ** (18,930 MESF/μm^2) and **LNA N1_FAM:15mQ** (14,050 MESF/μm^2).

(a)

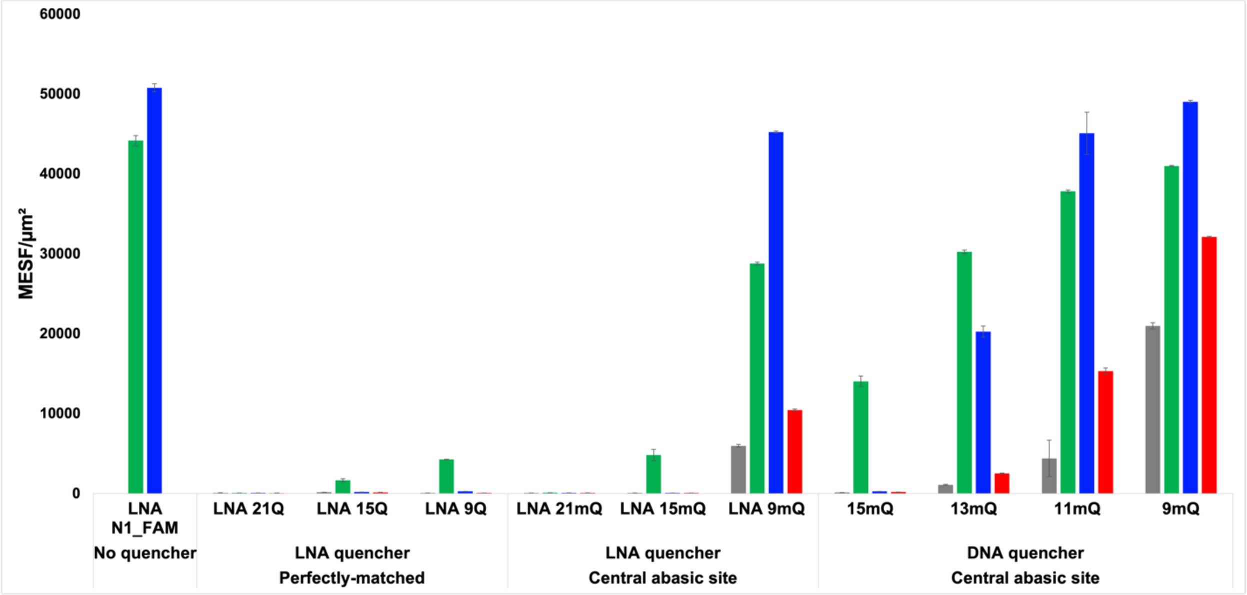

#3

(b)

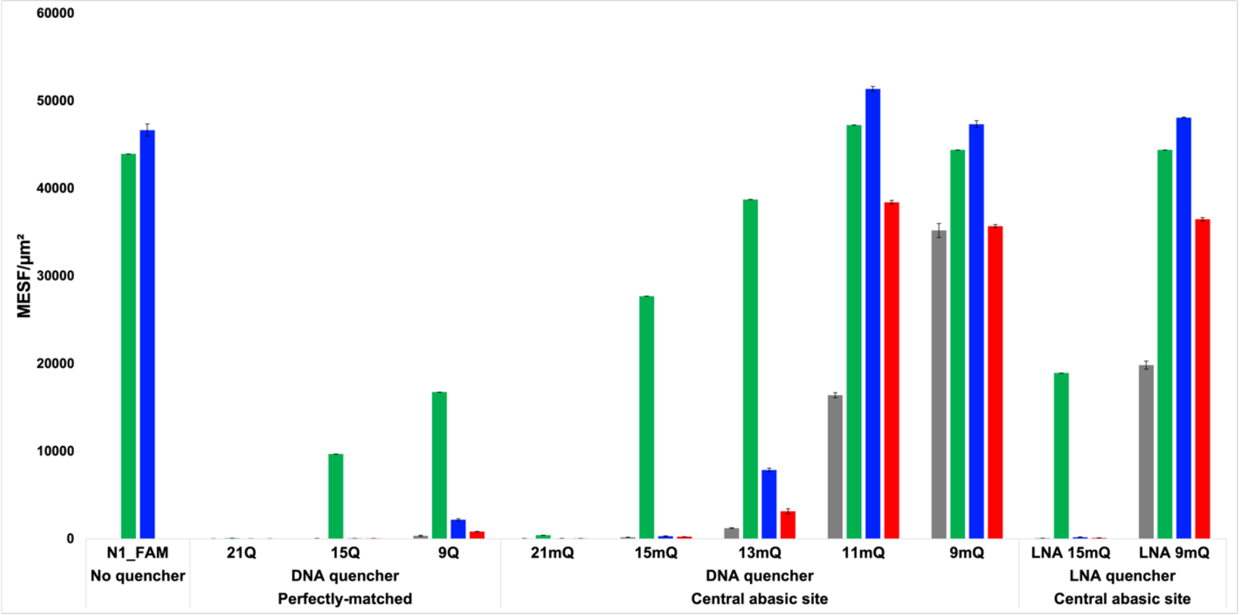

#1 #2

Figure S1. Bar graphs of normalized molecules of equivalent soluble fluorochrome (MESF) of **(a) LNA N1_FAM** and **(b) N1_FAM** probe systems prior to RNA addition (gray series) and following the addition of **SARS-CoV-2 RNA** (green series), **var_RNA** (blue series), or **scr_RNA** sequence (red series) for 1.05 µm microspheres (note: the studies described in the main article used 3.00 µm microspheres). Each sample was performed in triplicate and error bars indicate standard deviation values. Based on their selective response to **SARS-CoV-2 RNA**, the three dsprobe systems yielding highest fluorescence measurements are marked as #1, #2, and #3, respectively, below each probe's nomenclature in the bar graphs above.

Experimental Details

Sensitivity limit in select dsprobe system

N1_FAM and **15mQ** were combined in a 1:2 ratio and diluted in TE pH 8.0 to a final concentration of 5 μM **N1_FAM**. The mixture was annealed by heating to 94 ºC for 2 min, then slowly cooling to room temperature. A working solution of microspheres was prepared by diluting 5 μL bead stock from 1% w/v to 0.1% w/v in wash buffer. Microspheres were washed 3 times by centrifuging at 14,000*g* for 3 min, aspirating supernatant, then resuspending in fresh wash buffer. Pre-washed 3.00 μm microspheres were incubated with the pre-annealed dsprobe mixture at a final concentration of 1 μM **N1_FAM** and 0.1% w/v microspheres, and agitated for 15 min at 22ºC, 800 rpm on a thermomixer. The microspheres were then washed 3 times as described above. Following the final wash, the coupled microspheres were diluted 1000-fold to a final concentration of 0.0001% w/v and incubated with **SARS-CoV-2 RNA** at final concentrations ranging from 1 μM down to 100 pM, and agitated for 15 min at 22 ºC, 800 rpm on a thermomixer. For flow cytometry, 20 μL was taken from each sample and added to 400 μL PBS.

Separate quantification of primary duplex formation and extent of 15m displacement by RNA

Unlabeled **N1** and **15m_FAM** were combined in a 1:2 ratio and diluted in TE pH 8.0 to a final concentration of 5 μM **N1**. The mixture was annealed by heating to 94 ºC for 2 min, then slowly cooling to room temperature. Working solution of microspheres was prepared by diluting 5 μL bead stock from 1% w/v to 0.1% w/v in wash buffer. Microspheres were washed 3 times by centrifuging at 14,000*g* for 3 min, aspirating supernatant, then resuspending in fresh wash buffer. Pre-washed 3.00 μm microspheres were incubated with the pre-annealed dsprobe mixture at a final concentration of 1 μM **N1** and 0.1% w/v microspheres, and agitated for 15 min at 22ºC, 800 rpm on a thermomixer. The microspheres were then washed 3 times as described above. 5 μL was taken from each triplicate sample of coupled-microspheres to be used as the pre-RNA sample for background fluorescence. The rest of each triplicate was split to be incubated with one of three different RNA solutions: **SARS-CoV-2 RNA**, **var_RNA**, or **scr_RNA**, each at a final concentration of 1 μM RNA and 0.1% w/v microspheres. Mixtures were agitated for 15 min at 22 ºC, 800 rpm on a thermomixer, then 5 μL was removed from each and added to 400 μL PBS for flow cytometry.

Titration experiments to determine if self-quenching of immobilized FAM occurs

For the experiments depicted in Figure 4(a), varying ratios of **N1** to **N1_FAM** were diluted in TE pH 8.0 to a final concentration of 5 uM. For the experiment depicted in Figure 4(c), varying ratios of **N1** to **N1_FAM** were combined in a 1:2 ratio with **15m_noQ** and diluted in TE pH 8.0 to a final concentration of 5 μM probe. All mixtures were annealed by heating to 94 ºC for 2 min, then slowly cooling to room temperature. Working solution of microspheres was prepared by diluting 5 μL bead stock from 1% w/v to 0.1% w/v in wash buffer. Microspheres were washed 3 times by centrifuging at 14,000*g* for 3 min, aspirating supernatant, then resuspending in fresh wash buffer. Pre-washed 3.00 μm microspheres were incubated with the pre-annealed solutions at a final concentration of 1 μM probe and 0.1% w/v microspheres, and agitated for 15 min at 22ºC, 800 rpm on a thermomixer. The microspheres were then washed 3 times as described above. 5 μL was taken from each triplicate sample of coupled-microspheres to be used as the pre-RNA sample for background fluorescence. The rest of each triplicate was split to be incubated with one of three different RNA solutions: **SARS-CoV-2 RNA**, **var_RNA**, or **scr_RNA**, each at a final concentration of 1 μM RNA and 0.1% w/v microspheres. Mixtures were agitated for 15 min at 22 ºC, 800 rpm on a thermomixer, then 5 μL was removed from each and added to 400 μL PBS for flow cytometry.

Titration experiments to determine if residual quencher species has longer range effects on neighboring FAM moieties

For the experiment depicted in Figure 5(a), varying ratios of **15m** to **15mQ** were diluted in TE pH 8.0, and mixed with **N1_FAM** at a ratio of 2:1, with a final **N1_FAM** concentration of 5 μM. These mixtures were annealed by heating to 94 ºC for 2 min, then slowly cooling to room temperature. Working solution of microspheres was prepared by diluting 5 μL bead stock from 1% w/v to 0.1% w/v in wash buffer.

Microspheres were washed 3 times by centrifuging at 14,000*g* for 3 min, aspirating supernatant, then resuspending in fresh wash buffer. Pre-washed 3.00 µm microspheres were incubated with the pre-annealed solutions at a final concentration of 1 µM probe and 0.1% w/v microspheres, and agitated for 15 min at 22ºC, 800 rpm on a thermomixer. The microspheres were then washed 3 times as described above. 5 µL was taken from each triplicate sample of coupled-microspheres to be used as the pre-RNA sample for background fluorescence. The rest of each triplicate was split to be incubated with one of two different RNA solutions: **SARS-CoV-2 RNA** or **var_RNA** each at a final concentration of 1 µM RNA and 0.1% w/v microspheres. Mixtures were agitated for 15 min at 22 ºC, 800 rpm on a thermomixer, then 5 µL was removed from each and added to 400 µL PBS for flow cytometry.

For the experiments depicted in Figure 6(a), varying ratios of **15_noQ** to **15Q** were diluted in TE pH 8.0, and mixed with **N1** at a ratio of 2:1, with a final **N1** concentration of 5 µM. Separately, **B15** (5' - ATC AGC CGC AAT CCA – 3') and **A24_FAM** (5' - biotin - TTT TTT TTT TGG ATT GCG GCT GAT – FAM -3') were mixed at a ratio of 2:1, with a final **A24_FAM** concentration of 5 µM. All of these mixtures were annealed by heating to 94 ºC for 2 min, then slowly cooling to room temperature. Then, each **N1**:**15_noQ**/**15Q** sample was mixed with the **A24_FAM**:**B15** mixture described above, in a 1:1 ratio of **N1** to **A24_FAM**. Working solution of microspheres was prepared by diluting 5 µL bead stock from 1% w/v to 0.1% w/v in wash buffer. Microspheres were washed 3 times by centrifuging at 14,000*g* for 3 min, aspirating supernatant, then resuspending in fresh wash buffer. Pre-washed 3.00 µm microspheres were incubated with the pre-annealed solutions at a final concentration of 1 µM probe and 0.1% w/v microspheres, and agitated for 15 min at 22ºC, 800 rpm on a thermomixer. The microspheres were then washed 3 times as described above, then 5 µL was removed from each and added to 400 µL PBS for flow cytometry.

End-to-end length calculations to estimate separation distance range of various probe combinations

Scheme S1(a) shows the various possible combinations of immobilized probe and hybridization partner and the calculated end-to-end length of each. The ssDNA probe was modeled as flexible 24 base-long ssDNA; the dsDNA probe was modeled as a rigid 15 base-long dsDNA B-helix segment + a flexible 9 base-long ssDNA segment; the dsDNA:RNA was modeled as a rigid 24 base-long dsDNA:RNA A-helix; and the imperfect dsDNA:RNA was modeled as a rigid 11 base-long dsDNA:RNA A-helix segment + a semi-rigid 13 base-long dsDNA:RNA A-helix per Sim *et al.*[7]

Scheme S1(b) shows estimated separation distances between upright pairs of heterogeneous nearest neighbor oligonucleotides. The estimated separation distance is based on the reported[8] 2 nm separation distance between neighboring biotin-binding pockets on a given face of tetrameric streptavidin (modeled as a rectangle with two pairs of biotin-binding pockets on two opposing faces[8]) and theoretical segment lengths[7,9] of unhybridized and hybridized DNA and RNA as illustrated in Scheme S1(a).

(a)

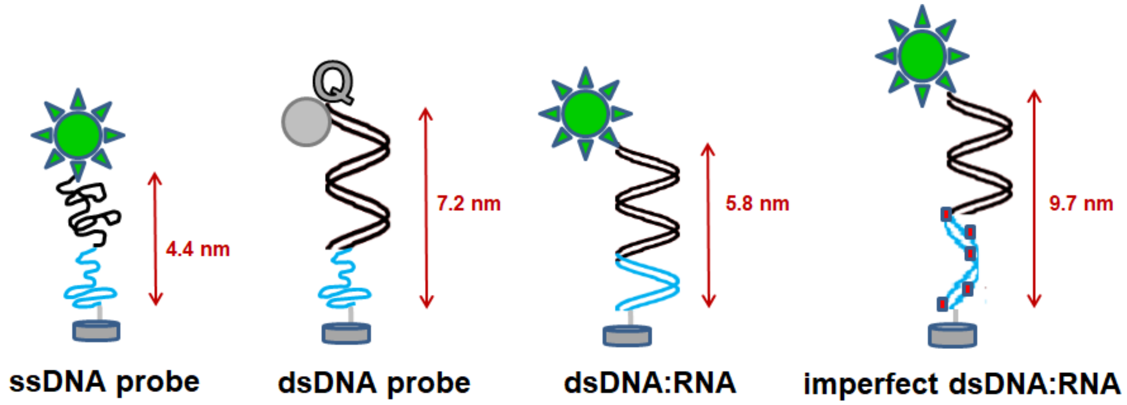

(b)

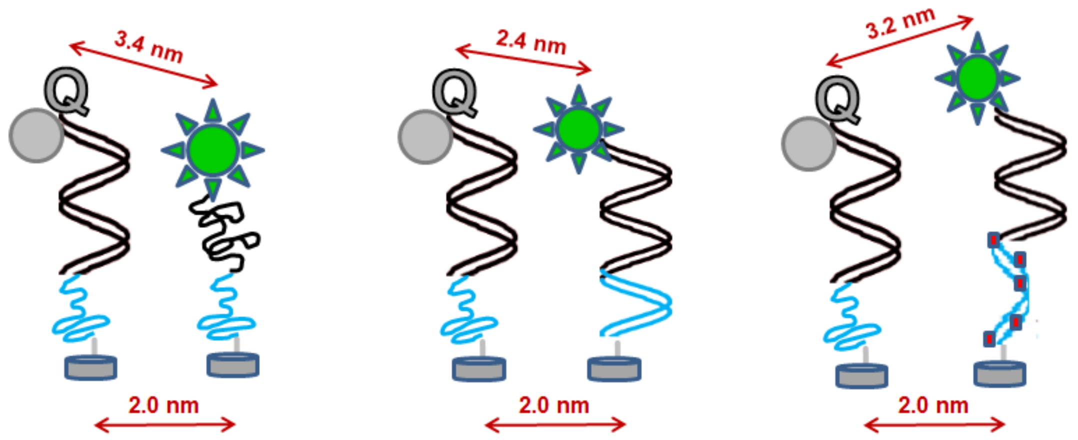

Scheme S1. (a) Various combinations of an individual **N1_FAM** DNA probe sequence unhybridized (*left*) and hybridized to a 15 base-long quencher-capped DNA sequence (*middle left*); a perfectly-matched RNA sequence (*middle right*); and an imperfectly-matched RNA variant sequence (*right*), each labeled with their respective, calculated end-to-end lengths. **(b)** Examples of pairwise heterogeneous combinations of a dsDNA (i.e., **N1_FAM:15mQ**) with a neigboring unhybridized **N1_FAM** (*left*); hybridized to perfectly-matched RNA sequence (*middle*); hybridized to imperfectly-matched RNA sequence (*right*). Each pair of neighboring oligonucleotides is labeled below with the 2.0 nm spacing between biotin binding sites on the same face of streptavidin and above with the estimated separation distance between two upright neighboring oligonucleotides. Though not illustrated in (**b**) the estimated separation distance between nearest neighbor ssDNA probe and imperfect dsDNA:RNA is 5.5 nm**.**

Effect of hybridization partner on N1_FAM fluorescence

In order to study the effects of different hybridization partners on the fluorescence of **N1_FAM**, microspheres coupled with **N1_FAM** were incubated with several different unlabeled RNA sequences. As shown in Figure S2, there was a slight decrease in fluorescence signal with the addition of **SARS-CoV-2 RNA**, but there was an increase in fluorescence signal with the addition of a truncated version of the sequence, **SARS-CoV-2(15)**. The addition of several variant RNAs (**var**, **var_0**, and **var_3**) also induced a higher fluorescence signal, but not uniformly. These results indicate that the differences in fluorescence signal of these various **N1_FAM:**RNA duplexes were induced by the base-length and fidelity of the unlabeled hybridization partner.

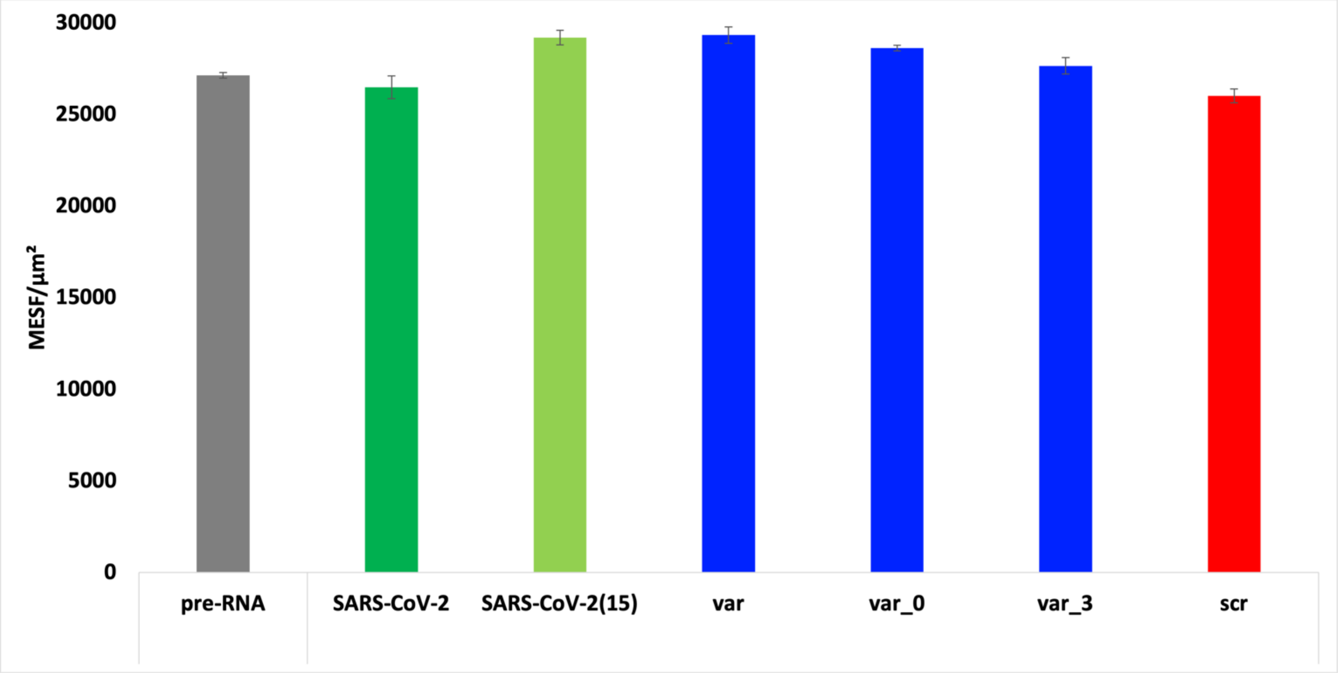

Figure S2. Bar graph of normalized molecules of equivalent soluble fluorochrome (MESF) of ssDNA **N1_FAM** probe systems prior to unlabeled RNA addition (gray) and following the addition of **SARS-CoV-2** (green), 15-base **SARS-CoV-2** (light green), model variant sequence mixture, **var**, and specific variant sequences, **var_0** and **var_3**, (blue series), or **scr** sequence (red series). Each sample was performed in triplicate and error bars indicate standard deviation values. For simplicity, the term "RNA" is excluded in the sequence nomenclature in both the bar graph x-axis as well as the figure caption.

References

1. Eze, N. A.; Milam, V. T., Quantitative analysis of in situ locked nucleic acid and DNA competitive displacement events on microspheres. *Langmuir* **2022,** *38* (22), 6871-6881. DOI: 10.1021/acs.langmuir.2c00220
2. Jensen, G.A., S.K. Singh, R. Kumar, J. Wengel and J.P. Jacobsen, A comparison of the solution structures of an LNA : DNA duplex and the unmodified DNA : DNA duplex. *J. Chem. Soc. Perkin Trans*. 2 **2001**(7) 1224-1232 DOI: 10.1039/b008431j
3. Vester, B. and J. Wengel, LNA (locked nucleic acid): high-affinity targeting of complementary RNA and DNA. *Biochemistry* **2004** 43(42) 13233-41 DOI: 10.1021/bi0485732
4. Mohrle, B.P., M. Kumpf and G. Gauglitz, Determination of affinity constants of locked nucleic acid (LNA) and DNA duplex formation using label free sensor technology. *Analyst* **2005** 130(12) 1634-1638 DOI: 10.1039/B507728A
5. Owczarzy, R., Y. You, C.L. Groth and A.V. Tataurov, Stability and mismatch discrimination of locked nucleic acid-DNA duplexes. *Biochemistry* **2011** 50(43) 9352-9367 DOI: 10.1021/bi200904e
6. Eze, N.A., R.S. Sullivan and V.T. Milam, Analysis of in situ LNA and DNA hybridization events on microspheres. *Biomacromolecules* **2017** 18 1086-1096 DOI: 10.1021/acs.biomac.6b01373
7. Sim, A. Y. L.; Lipfert, J.; Herschlag, D.; Doniach, S., Salt dependence of the radius of gyration and flexibility of single-stranded DNA in solution probed by small-angle x-ray scattering. *Physical Review E* **2012**, 86 (2), 021901. DOI: 10.1103/PhysRevE.86.021901
8. Ren, C.-l.; Carvajal, D.; Shull, K. R.; Szleifer, I., Streptavidin−biotin binding in the presence of a polymer spacer. A theoretical description. *Langmuir* **2009,** *25* (20), 12283-12292. DOI: 10.1021/la901735d
9. Shaw, N. N.; Arya, D. P., Recognition of the unique structure of DNA:RNA hybrids. *Biochimie* **2008,** *90* (7), 1026-1039. DOI: 10.1016/j.biochi.2008.04.011
